# Maryland Clinical Society

**Published:** 1894-12

**Authors:** H. O. Reik

**Affiliations:** No. 525 N. Howard St.; Baltimore, Md.


### Society Reports.

### MARYLAND CLINICAL SOCIETY.

### Stated Meeting held October 19th.

Dr. Randolph Winslow presented two cases of gastrotomy for cicatricial stenosis of the pharynx and œsophagus. The first case had been shown before the Society in the spring, and it was only exhibited again in order that the members might see the condition of the patient after the lapse of six months. The boy had not only been kept alive, but had grown fatter and stronger. He was fed regularly every four hours through the fistula—his diet consisting of milk, eggs, whiskey and cod-liver oil. He was also allowed to swallow as much milk as he was able to, but that was not a large quantity. The fistula had been in every way a success; it had saved him from impending starvation, it did not leak, and the surrounding tissues were entirely healthy.

The second case was of a similar nature to the first, a cicatricial contraction of the gullet from the ingestion of concentrated lye. The patient, a colored boy, one year and nine months of age was brought to the University Hospital, on July 11th, 1894. He had been healthy until six weeks before admission, when he swallowed some concentrated lye, the exact amount not having been ascertained. Until eight days before coming to the Hospital, he was able to drink a little milk, but since that time had not swallowed anything. Nutrient enemeta were ordered for him and on July 13th, Dr. Winslow performed gastrotomy by Frank's method. As he had had no nourishment for ten days, his stomach was opened at once and milk introduced. This was a mistake as some of the milk got on the wound and infection followed, with formation of a superficia abscess. His temperature which had run up to 104 as the result of this abscess, fell at once on the removal of a few stitches and the evacuation of a little pus. The patient had done well, but the fistula did not retain the contents of the stomach as perfectly as in the first case, probably on account of his incessant fretting and crying which caused prolapse of the mucous membrane of the stomach through the wound. These two boys have been saved from starvation by the performance of gastrotomy, but what is to become of them when they return to their homes? Their parents cannot or will not pay proper attention to them, and they will be likely to perish from neglect. Up to this time it has not been possible to dilate their strictures from above, and Dr. Winslow is contemplating reopening their stomachs and attempting dilation from below.

Dr. Rohe: I had a case about three years ago; a child had an attack of scarlet fever, after which there was a gradual closing of the œsophagus, until finally, nothing would pass into the stomach. At the Hopkins Hospital an attempt was made to dilate the œsophagus without success, and finally gastrotomy was advised. The child's mother objected, and left the hospital. Two weeks later he was brought again to the Maryland Hospital. No fluid would pass nor could any instrument be introduced. I tried electrolosis and feeding and for over a year, he was thus kept alive, but the contraction again closed, and finally he died. I found a small opening at the lower end of the œsophagus which might have been dilated from below. Electrolosis undoubtedly did good in this case, for his physical condition when first seen, was very poor, but during the year he had gained 25 pounds.

Dr. Julius Friedenwald read a paper on the use of the Resorcin test for the detection of free hydrochloric acid in the gastric juice.

Dr. I. R. Trimble read a paper entitled "Two Cases of Hæmaturia cured by Operation (Nephrectomy)."

Since the meeting of the Society, at which these cases were exhibited, Dr. Winslow has succeeded in passing a small bougie through the stricture in the second case, and making it protrude through the fistula, to which he tied a silk ligature and drew it upwards into the mouth. He used this as a saw after the manner of Abbe, and succeeded in dividing the stricture sufficiently for a No. 21 œsophageal bougie to pass.

STATED MEETING HELD FRIDAY, NOVEMBER 2d.

Dr S. C. Chew read a paper on "The Prevailing Epidemic of Typhoid Fever:"

I desire to call attention to some points connected with the prevailing epidemic of the disease, to seek to elicit opinions upon these points from my professional brothers present, and to see how fully they are consonant with or in opposition to my own. The points to be referred to are: First, the frequency and prevalence of the disease of late and at present, as compared with other recent years; second, the gravity of the cases as generally observed and on an average; third, the probable or possible causes of the prevailing epidemic so far as they can be traced.

First, as to the frequency. Judging from the number of cases I have met with in my own practice or in consultation with others during the past six months, I should be disposed to say that it is decidedly more prevalent than I have observed in this same space of time in any recent year. Again, the careful study of the Health Report for the past three or four years, brings one to the same conclusion.

Second, the gravity. As in previous years, I have found very different degrees of severity in my cases this time. Of thirty-three cases encountered in the past six months and in which the diagnosis of typhoid was perfectly clear, only one terminated fatally, death being due to repeated hemorrhage (intestinal). Two of the cases were so dangerously and critically ill that for several days the chances of death seemed greatly to preponderate over those for recovery. These two cases occurred in different parts of the city—a month intervening between their attacks. Of the remaining thirty cases, none, at least during the time that I saw some of them—and none at any time in my own cases—were critically ill, and I am strongly disposed to attribute the comparative mildness of a number of them to the early and assiduous use of the cold bath.

Third, the study of the causation of the present prevaence of typhoid fever, and the question whether there is any one cause that has been specially operative in producing it, presents great difficulties as is always the case when the disease occurs in an extensive community. I do not, of course, refer to the question of its microbic origin, for that is no longer *sub judice*, but I mean that the inquiry as to the manner in which the poison gains access to the system of patients is beset with great difficulties. The best opinions seem to favor the view that the chief medium of infection consists of liquid ingesta, chiefly water and sometimes milk—though with reference to the latter, it may be sometimes doubted whether it is the milk itself or the water with which it has been diluted, or in some way mixed. It is perhaps possible that the microbe of the disease may sometimes enter the system by inspired air, though that is thought very doubtful by some good authorities. And at any rate, the chief channel of invasion is the alimentary canal. The difficulty of determining the source of infection is illustrated by some facts that I will present: In an institution for the care of orphan girls in this city, an outbreak of typhoid fever occurred in September of this year, case following case in such rapid succession that in a short time twelve children out of a quota of 32 were attacked with the disease. Simultaneously with these cases, in a different part of the city and in another institution where boys and girls were received, the disease broke out and twelve of the inmates were attacked with it. Was there any factor common to these two groups of cases which may explain both of them? It is hard to believe that the general water supply of the city could be the special cause, for if it acted with such intensity and concentration in these two places, similar aggregations of cases would most probably be met with elsewhere. Could the milk supply of the two institutions account for both sets of cases? In both the milk supply was received from what was apparently the same source, which we may call Dairy "A." I have been assured by my friend, Dr. Green, who attended the cases in the first-named institution that no new cases occurred among the inmates after the process of boiling the milk and water was begun. This certainly gave ground for just suspicion, which was partly intensified when it was ascertained that the same dairy supplied the other institution. But this well-grounded suspicion was very much weakened when it was ascertained that the boys being housed in one building of that institution and the girls in another—the same milk being supplied to both—the girls were attacked by the disease while the boys escaped. This looked very much as if some other cause were operative; hence the case against the milk, while yet not deserving a verdict of not guilty was at least entitled to the Scotch verdict of not proven.

Dr. Osler: I have noticed in my hospital a much greater prevalence of disease this year than last. In last year, eighty cases; this year, sixty-one cases since the 15th of May, a considerable increase over corresponding dates of last year. I do not think our cases have been exceptionally severe. Of twenty cases I have under observation now, only one is critically ill.

Baltimore is in the position of a city that must be of necessity a typhoid city; that is always so of a city that has no sewerage system. A very proportional number of cases seen in the city come from outside districts. One of the most important points of danger I think is the milk supply, over which we seem to have no control. I do not think the death rate of typhoid fever will ever be at the minimum until the sewerage is changed and the milk supply brought under the proper control. Just as soon as this is done, supposing we have a good water supply, the death rate of Baltimore will be lowered.

Dr. McShane: Our water supply is not as bad as supposed; I heartily endorse what Dr. Osler has said, however. In the Annex and outlying districts of the city, the water is taken from wells and in 90 per cent. of these wells, the water is unfit for use.

The introduction of pipe to carry off the overflow from pits is simply a subterfuge. Nearly every pit in Baltimore is pervious and the soil is saturated with filth and this may be one of the means of contaminating the water.

Dr. Rohe: Anybody who tries to find the source of all cases of typhoid fever in one thing will make a mistake. The larger proportion of our cases in the city are probably brought home from resorts and outlying districts where well water is used. In another set of cases the water supply is that of the city, but even if you go to the source of supply and examine it, you will not find the germ, the cause being somewhere between the beginning of the supply and where it enters the house. If you have a direct continuation from the pipe out doors into the house, all is right, but if you draw water from an outdoor hydrant, which is close to the pit, it is different, the soil in filtration polluting the water in the hydrant box, which is apt to be pervious.

Dr. O'Donovan: To my mind it is far more important to look after the water supply of large cities. In Germany, the disease has been driven away, and should be here. Knowing these things, what should be said of us for drawing our supply from an open sewer, for that is what Lake Roland is. The water which flows into it comes very largely from the region of Towson whose streams are simply filthy. Should we allow to occur on the Gunpowder what we have seen occur in Lake Roland, when we know that such contamination can be kept down? The sewerage along these streams is washed with every rain into the river. The cause appears to be a practically avoidable one and the power to remove it should be not only given, but used, intelligently.

H. O. REIK, M. A.,
*No. 525 N. Howard St.* Baltimore, Md.

